# Integrated System of Solar Cells with Hierarchical NiCo_2_O_4_ Battery-Supercapacitor Hybrid Devices for Self-Driving Light-Emitting Diodes

**DOI:** 10.1007/s40820-019-0274-0

**Published:** 2019-05-22

**Authors:** Yuliang Yuan, Yangdan Lu, Bei-Er Jia, Haichao Tang, Lingxiang Chen, Yu-Jia Zeng, Yang Hou, Qinghua Zhang, Qinggang He, Lei Jiao, Jianxing Leng, Zhizhen Ye, Jianguo Lu

**Affiliations:** 1 State Key Laboratory of Silicon Materials, School of Materials Science and Engineering, Zhejiang University, Hangzhou, 310027 People’s Republic of China; 2 Key Laboratory for Biomedical Engineering of Ministry of Education, College of Biomedical Engineering and Instrument Science, Zhejiang University, Hangzhou, 310027 People’s Republic of China; 3 Shenzhen Key Laboratory of Laser Engineering, College of Optoelectronic Engineering, Shenzhen University, Shenzhen, 518060 People’s Republic of China; 4 College of Chemical and Biological Engineering, Zhejiang University, Hangzhou, 310027 People’s Republic of China; 5 Ningbo Research Institute, Zhejiang University, Ningbo, 315100 People’s Republic of China; 6 Ocean College, Zhejiang University, Zhoushan, 316021 People’s Republic of China

**Keywords:** Integrated system, NiCo_2_O_4_, Battery-supercapacitor hybrid devices, Self-driving, LED

## Abstract

An integrated system has been provided with a-Si/H solar cells as energy conversion device, NiCo_2_O_4_ battery-supercapacitor hybrid (BSH) as energy storage device, and light emitting diodes (LEDs) as energy utilization device. By designing three-dimensional hierarchical NiCo_2_O_4_ arrays as faradic electrode, with capacitive electrode of active carbon (AC), BSHs were assembled with energy density of 16.6 Wh kg^–1^, power density of 7285 W kg^–1^, long-term stability with 100% retention after 15,000 cycles, and rather low self-discharge. The NiCo_2_O_4_//AC BSH was charged to 1.6 V in 1 s by solar cells and acted as reliable sources for powering LEDs. The integrated system is rational for operation, having an overall efficiency of 8.1% with storage efficiency of 74.24%. The integrated system demonstrates a stable solar power conversion, outstanding energy storage behavior, and reliable light emitting. Our study offers a precious strategy to design a self-driven integrated system for highly efficient energy utilization.

**Electronic supplementary material:**

The online version of this article (10.1007/s40820-019-0274-0) contains supplementary material, which is available to authorized users.

## Highlights

- Integration of solar cells, BSHs, and LEDs was developed for energy conversion, storage, and utilization in one system.
- NiCo_2_O_4_//AC BSHs were charged by a-Si/H solar cells for stably driving LEDs showing high performances.

**Electronic supplementary material:**

The online version of this article (10.1007/s40820-019-0274-0) contains supplementary material, which is available to authorized users.

## Graphical abstract

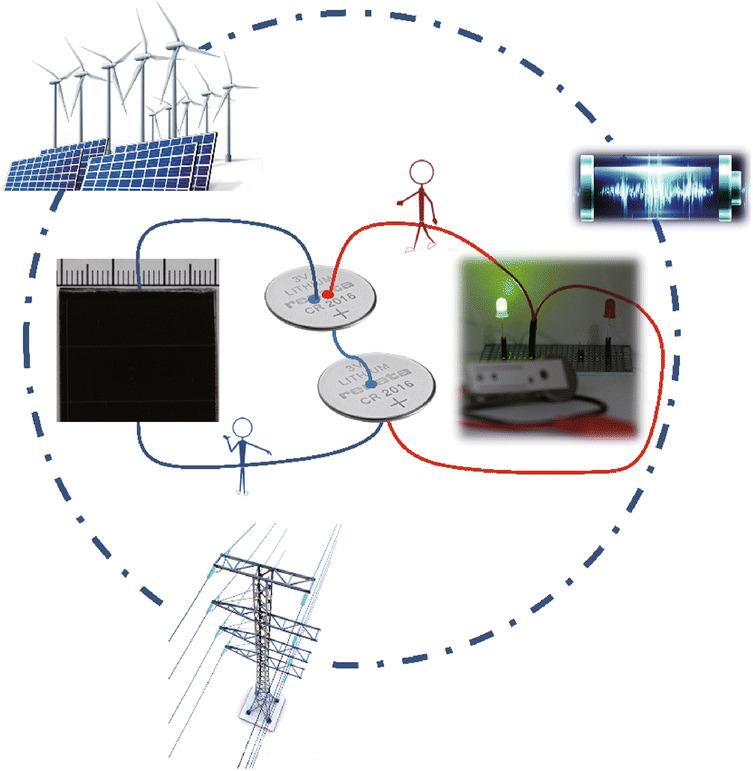

## Introduction

Combination of energy conversion, storage, and utilization devices has been widely investigated due to its possibilities in practical applications [1–3]. Among the three devices, electrochemical energy storage devices serve as the core component for both energy storage and power output [4]. As is well known, clean energies, including solar energy, wind energy, and tidal energy that are expected to be used in large scale in the near future, are intermittent and even irregular owing to the changes in weather and thus unsuitable for all day power supply [5]. For instance, solar energy can only work under sunlight illumination, which is affected by not only the cycle of day and night, but also the variation of sunlight intensity during daytime such as clear and cloudy. Therefore, the energy devices are indispensible to serve as a buffer pool to mitigate the mismatch between solar energy supply and demand.

Currently, electrochemical energy storage devices cover batteries and supercapacitors primarily [6–12]. Batteries are really a big family, including conventional lead–acid [13, 14], nickel–cadmium [15, 16], nickel–metal hydride [17–19], and lithium-ion batteries [20–25], as well as newly developed batteries such as lithium–sulfur [26–28], lithium–air [29–31], lithium–CO_2_ [32–34], sodium/potassium/magnesium/aluminum/zinc ion [35–44], and aqueous metal ion batteries [45, 46]. However, the batteries are unsuitable as energy storage devices in the frequently charge and discharge area, such as solar energy, wind energy, and tidal energy, due to the shortcomings of low cycling performance (usually in the order of hundreds of times) [47, 48]. Conventional supercapacitors possess the virtual of long cycling lifetime, but they are restricted to the low energy density and high self-discharge. The giant volume is inevitable to match the energy output, which prevents supercapacitors from practical use. Recently, a new analogous device combined with faradic electrode and capacitive electrode, named hybrid supercapacitor (HSC) or battery-supercapacitor hybrid (BSH) [49–52], provides a reliable approach to make the hybrid devices with advantages of both batteries and supercapacitors. This combination is promising to satisfy the requirements of both high energy density and high power density to drive different kinds of electrical equipments. With the merit of both long cycling characteristic and high energy density, the BSH devices are particularly suitable for the periodically changed energy generation sources.

In this work, we developed an integrated system with a-Si/H solar cells, three-dimensional (3D) hierarchical NiCo_2_O_4_ arrays//active carbon (AC) BSHs, and light-emitting diodes (LEDs) to realize energy conversion, storage, and utilization in one system. Benefitting from the unique hierarchical structure, the 3D hierarchical NiCo_2_O_4_ arrays electrode exhibits a high specific capacity of 130 mAh g^−1^. The BSHs assembled with NiCo_2_O_4_ faradic electrode and AC capacitive electrode display an energy density of 16.6 Wh kg^−1^, power density of 7,285 W kg^−1^, long cycling stability (100% retention after 15,000 cycles), and rather low self-discharge. Acting as the energy storage device in the integrated system, the BSHs can store solar energy and output power for driving LEDs. The NiCo_2_O_4_//AC BSHs are charged to 1.6 V in 1 s by the solar cells under light illumination, and then, the stored energy rationally powers the LEDs for light emitting. The self-driven integrated system has an overall efficiency of 8.1% with the storage efficiency of 74.24%. This study is expected to pave a road for commercial applications of BSHs in self-driven integrated systems. Moreover, the design of integrated system is expected to provide a potential strategy for clean energy applications, which is of great importance in the situation of today’s growing shortage of fossil energy.

## Experimental

### Synthesis of 3D Hierarchical NiCo_2_O_4_ Arrays

Hierarchical NiCo_2_O_4_ arrays were prepared by a continuous synthesis process. As the beginning step, a slice of Ni foam (20 × 40 × 1.8 mm^3^) was weighed for experiments. Nickel chloride hexahydrate (2.5 mmol), cobalt chloride hexahydrate (5 mmol), carbamide (9 mmol), and hexadecyl trimethyl ammonium bromide (CTAB, 2 mmol) were dissolved in ultrapure water (50 mL). After being stirred by magnetic stirrer to form homogeneous solution, the solution was transferred to a high-pressure autoclave with fill factor of 60%. The prepared Ni foam acted as substrate, and the reaction temperature was 100 °C for 6 h in an oven. Afterward, the Ni foam with products was rinsed with ultrapure water to remove any impurities and dried. After an annealing process at 350 °C for 3 h, the NiCo_2_O_4_ nanowire arrays were formed on the Ni foam, which are the intermediate product to obtain the final product.

A simple chemical bath deposition method was used for the further growth of NiCo_2_O_4_ nanoflakes. Nickel sulfate (1.0 M in 10 mL), cobaltous sulfate (2 M in 10 mL), and potassium peroxydisulfate (0.25 M in 16 mL) were mixed to form a solution, and then, the ammonia (25 − 28%, 4 mL) was added. The NiCo_2_O_4_ nanowire arrays grown on Ni foam were immersed into the obtained solution for 8 min to load NiCo_2_O_4_ nanoflakes. After the reaction, an annealed process (350 °C, 2 h) was carried out on the sample to achieve the final product of 3D hierarchical NiCo_2_O_4_ arrays. The mass loading of hierarchical NiCo_2_O_4_ arrays is ~ 4 mg cm^−2^.

### Assembly of NiCo_2_O_4_//AC BSHs

The BSH devices were assembled with NiCo_2_O_4_ hierarchical arrays as the faradic electrode and AC as the capacitive electrode. The filter paper and 3 M KOH solution were used as membrane and electrolyte, respectively. The AC capacitive electrode was prepared by a generic method. Specifically, the AC, acetylene black, and polyvinylidene fluoride were mixed with a mass ratio of 8:1:1 under vigorous stirring. Then, the *N*-methyl pyrrolidone was dropped into the mixture under stirring. The paste was coated on the Ni foam after homogeneously mixed. Finally, the faradic electrode, filter paper membrane, and capacitive electrode were encapsulated in a coin cell.

### Integration of Self-Powered System

**Fig. 1:**
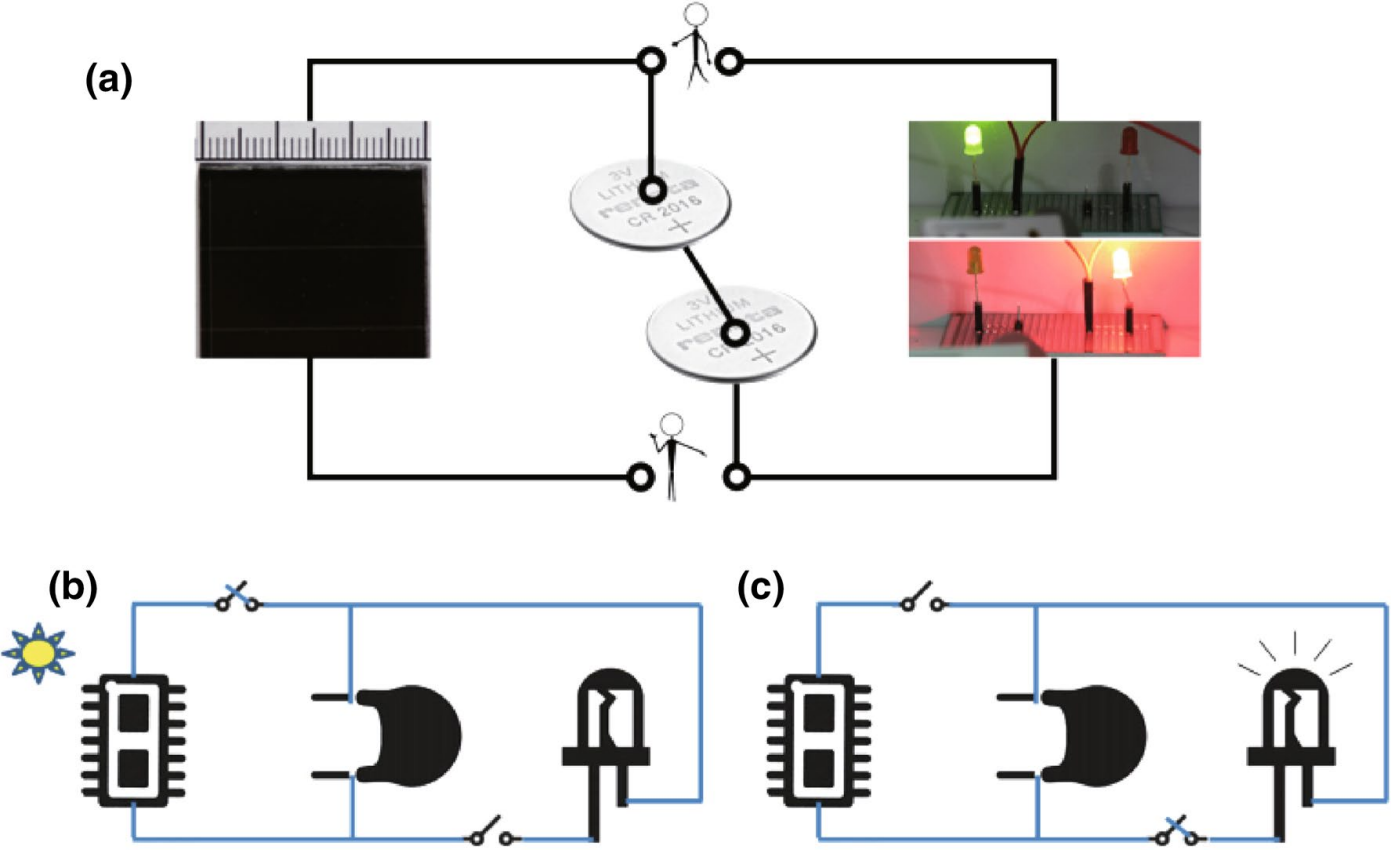
**a** Schematic of the integrated system consisting of a-Si/H solar cells, NiCo_2_O_4_//AC BSHs, and LEDs as the energy conversion, storage, and utilization devices. **b** Equivalent circuit describing the integrated system in the energy conversion and storage mode. **c** Equivalent circuit describing the integrated system in the energy supply and utilization mode

On the assemble of integrated hybrid system (Fig. 1), the a-Si/H solar cells was served as the energy conversion device, NiCo_2_O_4_ hierarchical arrays//AC BSHs as the energy storage device, and LEDs as the energy utilization device. They can be integrated into a module in practical. The single-junction a-Si/H solar cells were fabricated by ourselves, whose structure was designed as: glass/textured ZnO/Al (AZO)/p-type a-Si/H/intrinsic a-Si/H/n-type a-Si/H/Al [53]. Here, textured AZO films that are etched by a unique salt solution are adopted as the front contact layer. The procedures for fabricating a-Si/H solar cells were illustrated in our previous study [53]. The red and green LEDs were commercially obtained.

### Measurements and Evaluations

The characterizations of materials and performance evaluations of each device and whole integrated system are described in detail in Note S1 in the Supporting Information.

## Results and Discussion

Figure 1a displays the diagram of integrated system. In a typical workflow, the BSHs were charged by the a-Si/H solar cells and then powered the LEDs for light emitting. Figure 1b, c gives the equivalent circuit describing the circuit connection of solar cells, BSHs, and LED in parallel. Figure 1b shows the charge or energy storage process of BSHs by solar cells under light illumination without LEDs in circuit. Theoretically, the BSHs can be charged to the open-circuit voltage of solar cells. Figure 1c exhibits the discharge process of BSHs by output electric power for LEDs when light source is removed.

### Properties of 3D Hierarchical NiCo_2_O_4_ Arrays

**Fig. 2:**
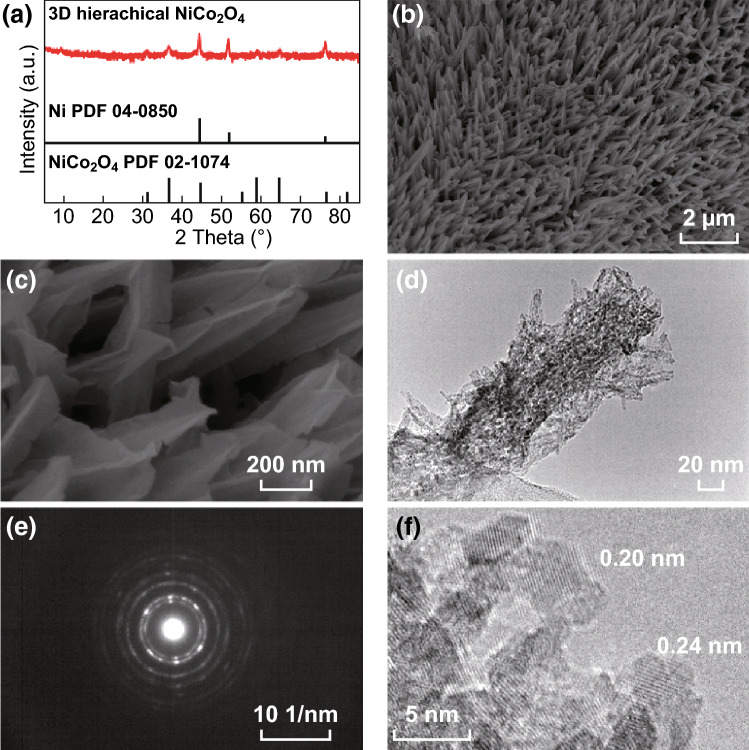
**a** Hierarchical NiCo_2_O_4_ arrays (red line) and two-stand diffraction pattern of metallic nickel and face-centered cubic NiCo_2_O_4_. SEM images of hierarchical NiCo_2_O_4_ arrays at **b** low and **c** high magnifications. **d** TEM image and **e** SAED pattern of hierarchical NiCo_2_O_4_ nanostructure. **f** Lattice diffraction fringes of NiCo_2_O_4_ recorded at HRTEM. (Color figure online)

For the assembly of BSHs, we have synthesized 3D hierarchical NiCo_2_O_4_ arrays by a continuous two-step solution process (as shown in Fig. S1), which acted as the faradic electrodes in the devices. The X-ray diffraction (XRD) patterns (Figs. 2a and S1d) and X-ray photoelectron spectroscopy (XPS) spectra (Fig. S2) confirm the formation of NiCo_2_O_4_ with face-centered cubic crystal. Figure 2b, c shows the scanning electron microscope (SEM) image of the product. It can be clearly seen that large-area 3D hierarchical NiCo_2_O_4_ arrays are uniformly formed. A NiCo_2_O_4_ hierarchical structure is composed of a core of NiCo_2_O_4_ nanowire and a shell of several NiCo_2_O_4_ nanoflakes, with the shape like a lengthened carambola. Each nanowire is fully covered by nanoflakes, and each NiCo_2_O_4_ nanoflake is parallel with the nanowire. In such a way, abundant nanochannels form between nanoflakes, which can form a fast infiltration path for electrolyte. The 3D hierarchical structure makes all nanowires and nanoflakes highly accessible to electrolyte for energy storage. Also, it is a smart way to increase the mass loading for improving the performance of electrodes.

Detailed structure and morphology of as-prepared 3D hierarchical NiCo_2_O_4_ arrays were further investigated by transmission electron microscope (TEM) as illustrated in Fig. 2d–f. Figure 2d displays the TEM image of an individual NiCo_2_O_4_ hierarchical nanostructure. The NiCo_2_O_4_ nanowire is elegantly wrapped with leaf-like NiCo_2_O_4_ nanoflakes, forming a hierarchical homostructure with enhanced surface area. Figure 2e exhibits the selected area electron diffraction (SAED) pattern of NiCo_2_O_4_ hierarchical structure. The concentric circles with a large amount of diffraction spots in SAED patterns can be obviously observed, which demonstrate that the NiCo_2_O_4_ hierarchical arrays are polycrystalline in nature. Figure 2f shows the high-resolution TEM (HRTEM) image of the NiCo_2_O_4_ nanoflake shell in one NiCo_2_O_4_ hierarchical structure, with planar spacings of 0.20 and 0.24 nm corresponding to (400) and (311) faces of cubic NiCo_2_O_4_.

**Fig. 3:**
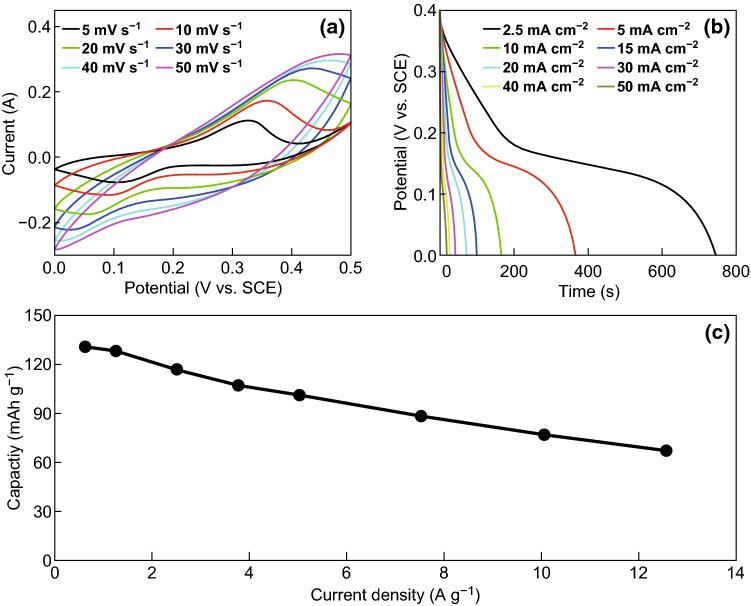
**a** CV curves of hierarchical NiCo_2_O_4_ electrode at various scan rates. **b** GCD curves and **c** specific capacity of hierarchical NiCo_2_O_4_ electrode at current densities from 0.63 to 12.56 A g^−1^

Electrochemical properties of 3D hierarchical NiCo_2_O_4_ arrays were first characterized by cyclic voltammetry (CV) in KOH solution with saturate calomel electrode (SCE) as reference electrode and Pt foil as counter electrode. Figure 3a depicts the CV curves of 3D hierarchical NiCo_2_O_4_ arrays at scan rates from 5 to 50 mV s^−1^ in the potential range of 0 − 0.5 V. The CV curves display a pair of redox peaks generated from positive and negative scan, confirming an obvious redox reaction on the electrode. Generally, this process corresponds to the redox process of Ni^2+^/Ni^3+^, Co^2+^/Co^3+^, and Co^3+^/Co^4+^ [54]. With the increase in scan rate, the redox peaks become less obvious because of a larger ratio of double-layer capacitance from faster scan rate. Clearly, the distinct redox peaks appear over the entire range of scan rates, demonstrating the rapid interfacial charge transport process and the electronic/ionic transport rate.

Galvanostatic charge discharge (GCD) properties of hierarchical NiCo_2_O_4_ electrodes were recorded at current densities from 0.63 to 12.56 A g^−1^, and the results are shown in Fig. 3b. The tilted platform during discharge illustrates a redox reaction during this process, in accordance with a redox reaction energy storage process. The specific capacity of hierarchical NiCo_2_O_4_ electrodes is calculated by using GCD curves. Figure 3c illustrates the derived specific capacity values. As the current density increases from 0.63 to 12.56 A g^−1^, the specific capacity of 3D hierarchical NiCo_2_O_4_ arrays decreases from 130 to 67 mAh g^−1^, with 51.4% capacity retained suggesting the high rate capability. All the values are remarkable for use in BSHs. The excellent electrochemical properties can be attributed to the sophisticated 3D hierarchical nanostructure with elevated mass loading, plenty of diffusion channels, and abundant pore structures, which increases the mass of active materials and provide enough active sites for interfacial reactions.

### Behaviors of Hierarchical NiCo_2_O_4_//AC BSHs

**Fig. 4:**
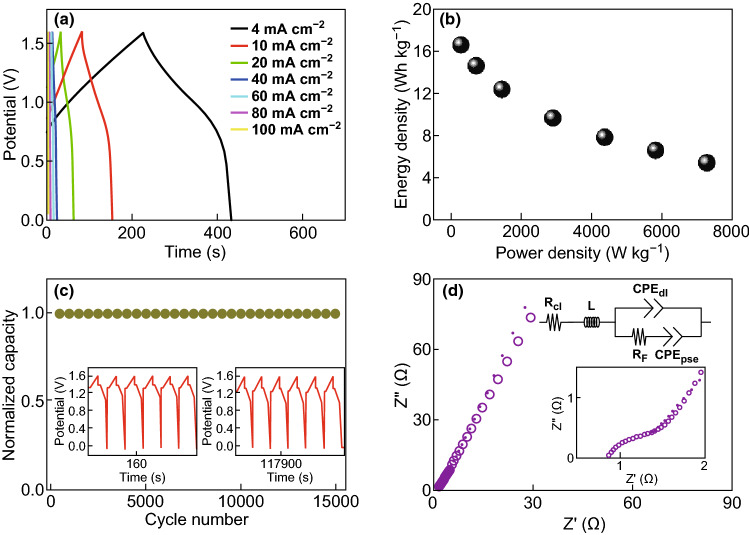
**a** GCD curves of NiCo_2_O_4_//AC BSH at current densities of 4–100 mA cm^−2^. **b** Ragone plot of BSH at different current densities. **c** Cycling stability of NiCo_2_O_4_//AC BSH at current densities of 20 mA cm^−2^. **d** EIS plot of BSH (The inset shows the enlarged part at high frequency region)

The BSH devices were assembled using 3D hierarchical NiCo_2_O_4_ arrays as the faradic electrode and AC as the capacitive electrode. No any binders were used in the NiCo_2_O_4_ cathode, which is of advantages for practical applications. The mass loadings of active materials were 1.1 and 4.4 mg for the NiCo_2_O_4_ and AC electrodes, respectively. Figure 4a illustrates the GCD curves of NiCo_2_O_4_//AC BSHs. The charge–discharge curves display neither an apparent platform in faradaic behavior nor a perfectly triangle shape in double-layer capacitor, suggesting that the BSHs are composed of two kind behaviors. Our BSHs can output a cell voltage of 1.6 V, as shown in Fig. 4a. The BSHs were fabricated in a CR2016 coin cell (diameter of 20 mm and thickness of 1.6 mm), as illustrated in Table S1. The Ragone plot (Fig. 4b) reveals the relation of energy density and power density of BSHs. The BSHs exhibit a high energy density of 16.6 Wh kg^−1^ at a power density of 291 W kg^−1^ and a high power density of 7,286 W kg^−1^ at an energy density of 5.5 Wh kg^−1^. The volumetric energy density and power density are provided in Fig. S3. The BSHs have a volumetric energy density of 1.14 mWh cm^−3^ at a volumetric power density of 20.04 mW cm^−3^ and a volumetric energy density of 0.38 mWh cm^−3^ at a volumetric power density of 500.91 mW cm^−3^.

Cycle performance is conducted for further evaluating BSHs (Fig. 4c). The BSH exhibits an excellent cycle performance with 100% retention of capacity after 15,000 cycles, demonstrating that the 3D hierarchical homostructure can meet the requirement of long cycle ability. The two insets in Fig. 4c show the GCD curves of the first six cycles and last six cycles. The unchanged shape of GCD curves from the beginning to the end confirms the long-term stability of our BSH. Also, an electrochemical kinetic analysis was carried out to provide a detail insight on energy storage mechanisms (Fig. S4). Both the double-layer capacity and faradic capacity contributed to the whole capacity.

Electrochemical impedance spectroscopy (EIS) of BSHs was further acquired under sinusoidal disturbance voltage from 1 Hz to 100 kHz (Fig. 4d). The equivalent circuit diagram is displayed in Fig. S5, where *R*_el_ represents solution resistance, *L* represents inductance, *CPE*_dl_ represents double-layer capacitance, *R*_F_ represents charge transfer resistance, and *CPE*_pse_ represents pseudocapacitance. The depressed semicircle is observed in the high-frequency area and the sloping line in the low-frequency area, revealing the combination of charge transfer resistance with capacitive features. The equivalent serial resistance (*R*_el_) obtained from the fitted data is only 0.74 Ω, illustrating a very low internal resistance of the BSHs device. The charge transfer resistance (*R*_F_), caused by the faradic reactions at the surface of electrode, is around 0.97 Ω. Bode graph of EIS plot is provided in Supplementary Material (Fig. S6), where the fitted curve matches well with the experimental one, confirming the rationality of fitting procedure. The impedance data verify that our BSHs possess both low internal resistance and low charge transfer resistance.

### Performances of Self-Driven Integrated System

**Fig. 5:**
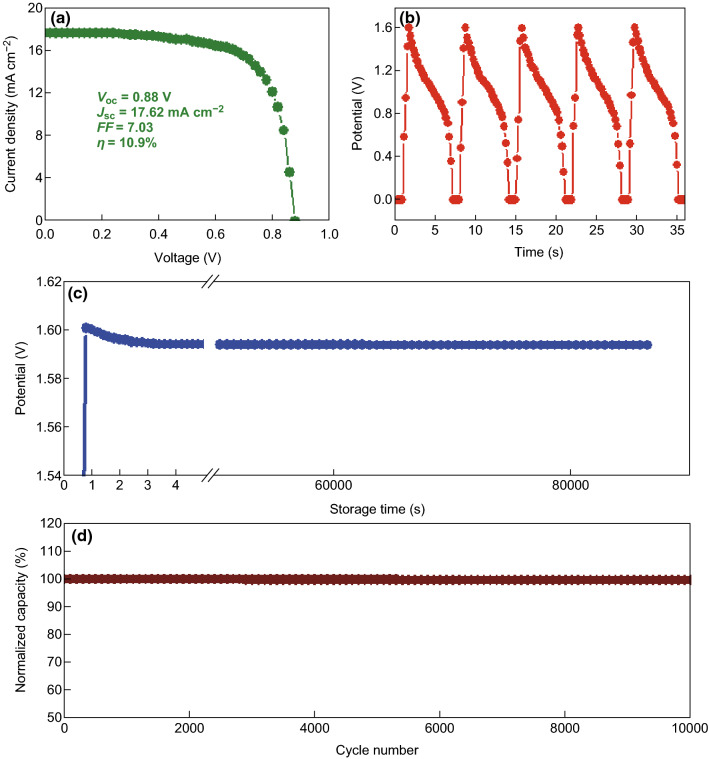
**a** *J*–*V* curve of single-junction a-Si/H solar cells. **b** Charge–discharge curve of the NiCo_2_O_4_//AC BSHs powered by the solar cells in the integrated system. **c** Self-discharge curve of NiCo_2_O_4_//AC BSH in the integrated system. **d** Cycling performance of NiCo_2_O_4_//AC BSH in the integrated system

Self-driven integrated system is designed by using NiCo_2_O_4_//AC BSH as the energy storage device. Single-junction a-Si/H solar cells are employed as the energy conversion device, and LEDs as the energy utilization device, as displayed in Fig. 1. The properties of a-Si/H solar cells were individually investigated in the first place. The photocurrent density–photovoltage (*J*–*V*) curves of the a-Si/H solar cells tested under AM 1.5 G illumination at 100 mW cm^−2^ are displayed in Fig. 5a. The open-circuit voltage (*V*_oc_), short-circuit current density (*J*_sc_), fill factor (*FF*), and conversion efficiency are determined to be 0.88 V, 17.62 mA cm^−2^, 0.703, and 10.9%, respectively. The solar cells we used in the experiment possesses exhibit almost the same performances as reported in our previous studies [53].

The a-Si/H solar cells serve as the energy production device in the integrated system under sunlight illumination. In the experiment of integrated system, the NiCo_2_O_4_//AC BSH was charged by two solar cells in series under AM 1.5 G irradiation. In Fig. 5b, the BSH can be charged readily by the solar cells (via solar cell charging controller) with a saturation voltage of 1.601 V due to the *V*_oc_ restriction. The charging process can be accomplished within 1 s. The NiCo_2_O_4_//AC BSHs was constantly discharged at 100 mA cm^−2^. Figure 5b provides five charge and discharge curves of the BSHs supplied by solar cells. All the five curves show exactly similar shape, illustrating the stable and reliable nature of energy production and storage.

Conventional supercapacitors generally have a tendency for relatively fast self-discharge rate due to the thermodynamic favorable characteristic. The self-discharge mechanisms mainly include overcharging, side reaction, and ohmic leakage. As a similar structure of BSHs with conventional supercapacitors, the self-discharge behavior of the BSHs was studied in our work after it was charged by the solar cells. After charging the BSHs up to specified potential, it was allowed to self-discharge in an open-circuit state, which was monitored with a voltmeter. The BSH self-discharge timescales and shapes in Fig. 5c illustrate an open-circuit voltage variation curve. From the curve, it can be seen that the voltage of the BSHs is first charged to 1.601 V by the solar cells and then decreases to 1.594 V in the first 5 s; afterward, the voltage kept unchanged in the rest time (in 24 h during measurements). These investigations verify the property of integrated hybrid system with a rather low self-discharge.

Cycling stability of the integrated system was also investigated by charging the BSHs with the solar cells, and then discharging galvanostatically for 10,000 cycles, as shown in Fig. 5d. After 10,000 charging and discharging cycles, the capacity of the BSH is decreased only 0.3%, suggesting the excellent cycling stability. The high cycling stability of BSHs driven by solar cells is comparable with that driven by electrochemical workstation. Despite the participation of faradic electrode during charge and discharge process, the high cycling stability is almost the same with double-layer supercapacitors. In the long cycling stability test up to 10,000 cycles, no obvious deterioration is observed, which is really important for BSHs in practical applications. To further confirm the stability of our BSHs, the voltage holding test was investigated at 1.6 V, as shown in Fig. S7. During a procedure for 100 h, the capacity retention was around 90.7% for the NiCo_2_O_4_//AC BSH, which can be comparable with previously reported in carbon-based electric double-layer capacitors [55, 56]. The long-lasting cycling stability of NiCo_2_O_4_//AC BSHs in our work is rarely obtained, which is believed to be attributed to the well-designed 3D hierarchical structure. The superior cycling performance of the NiCo_2_O_4_ array-based BSHs makes it suitable for energy storage of intermittent energies, including solar energy, wind energy, and tidal energy.

One important factor describing the performance of the integrated hybrid system is the overall energy conversion efficiency (*η*_overall_). The *η*_overall_ is defined by the ratio of effective output energy to the overall input energy. In the case of our integrated system, the effective output energy is defined by the discharge of 3D NiCo_2_O_4_ hierarchical arrays//AC BSHs; the overall input energy is defined by the illumination of AM 1.5 G. Thus, the overall energy conversion efficiency can be described as:

1 $$\eta = \frac{{I_{\text{dis}} \int v {\text{d}}t}}{PSt}$$

where *I*_dis_ represents the discharge current of 3D NiCo_2_O_4_ hierarchical arrays//AC BSH, *v* represents the cell potential during discharge, *P* represents power density of illumination light, *S* represents the light-sensing area of a-Si/H solar cells, and *t* represents illumination time. The *η*_overall_ determined using this equation is calculated to be 8.1%, which is certainly a reasonable result for our hybrid system with a slightly decrease, as compared with the conversion efficiency of 10.91% for a-Si/H solar cells. The overall conversion efficiency of 8.1% is high and commendable in photovoltage-energy storage systems, because the overall conversion efficiency is below 5% in most cases.

**Fig. 6:**
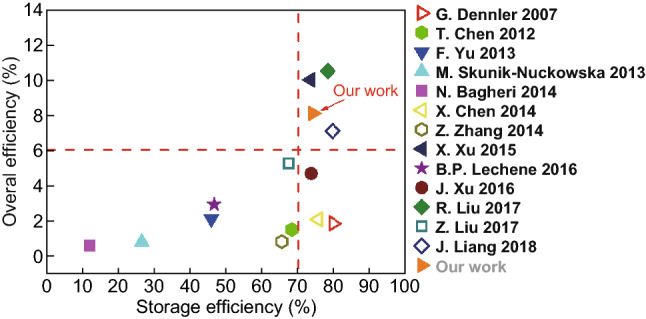
Overall efficiency and storage efficiency of photovoltage-energy storage systems in all the available studies reported in recent years. The details of references are listed in Table S2

The storage efficiency, defined as the efficiency of energy output from solar cells to BSHs, has also been taken into account. Table S2 lists the overall efficiency and storage efficiency of photovoltage-energy storage systems in almost all available studies reported in recent years. For clear identification, the overall efficiency and storage efficiency of available photovoltage-energy storage systems are plotted in Fig. 6. In the plot, the *x*-axis denotes storage efficiency, and the *y*-axis denotes overall efficiency. Thus, the obtained data located at the top-right corner signify both high overall efficiency and high storage efficiency. For our photovoltage-energy storage system, the overall efficiency is 8.1% and the storage efficiency is 74.24%, which is located at the top-right corner together with other three reported works, superior to most reported values, revealing the excellent performance of the system.

**Fig. 7:**
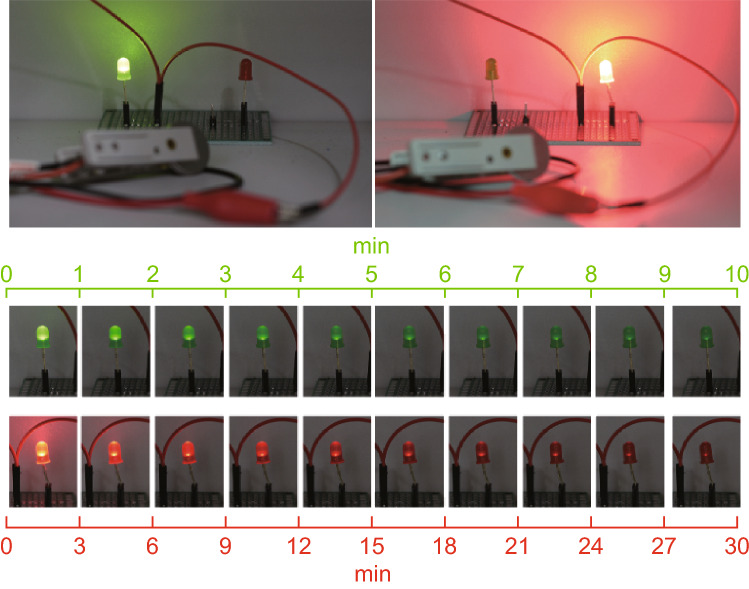
The photograph of green and red LEDs lit up by our BSHs and the light intensity variation of green LED in 10 min and red LED in 30 min in the integrated system. (Color figure online)

After being charged by the solar cells, the BSHs can serve as power sources for a variety of electrical devices. For example, the BSHs devices can rationally power various LEDs. Figure 7 gives the time-dependent brightness of green and red LEDs after being lit up by our BSHs. Since the coin BSHs are charged by the solar cells under illumination, they can power a green LED for more than 10 min, and a red LED for more than 30 min, respectively, which are applausive observations. This result strongly confirms the feasibility of our integrated device as self-powered systems.

## Conclusions

In conclusion, we have proposed an integration of energy conversion (a-Si/H solar cells), storage (NiCo_2_O_4_//AC BSHs), and utilization (LEDs) in one system. Each of the component functions well, and the whole system works independently. As the key of the integrated system, NiCo_2_O_4_//AC BSHs are elaborately designed, with lengthened carambola-like 3D hierarchical NiCo_2_O_4_ arrays as faradic electrodes and AC as capacitive electrodes. The BSHs exhibit excellent overall performances with high energy density of 16.6 Wh kg^−1^, high power density of 7,285 W kg^−1^, long cycling stability of 100% retention after 15,000 cycles, and rather low self-discharge. During the operation, the NiCo_2_O_4_//AC BSHs are charged to 1.6 V within 1 s by a-Si/H solar cells, serving as a stable voltage supply for powering LEDs. The self-driven integrated system has an overall efficiency of 8.1% with the storage efficiency of 74.24%, presenting stable and reliable behaviors in photovoltaic conversion, efficient energy storage, and functions as light emitting. This work is supposed to prove the potential applications of BSHs in self-driven systems and provide an evaluation method for such kind of integrated systems.

## Electronic supplementary material

Below is the link to the electronic supplementary material.

Supplementary material 1 (PDF 755 kb)
