# Supplementary material for: Integrated System of Solar Cells with Hierarchical NiCo2O4 Battery-Supercapacitor Hybrid Devices for Self-Driving Light-Emitting Diodes
Source: Nanomicro Lett. 2019 May 22;11:42. doi: 10.1007/s40820-019-0274-0 (PMC7770920; doi:10.1007/s40820-019-0274-0)
Supplement: Supplementary file 1 — Supplementary material 1 (PDF 755 kb) [file 40820_2019_274_MOESM1_ESM.pdf]

Supporting Information for

Integrated System of Solar Cells with Hierarchical $NiCo_2O_4$ Battery-Supercapacitor Hybrid Devices for Self-driving Light-emitting Diodes

Yuliang Yuan[1,2], Yangdan Lu[1], Bei-Er Jia[1], Haichao Tang[1], Lingxiang Chen[2], Yu-Jia Zeng[3], Yang Hou[4,5,*], Qinghua Zhang[4,5], Qinggang He[4,5], Lei Jiao[6], Jianxing Leng[6], Zhizhen Ye[1], Jianguo Lu[1,2,5,*]

[1]State Key Laboratory of Silicon Materials, School of Materials Science and Engineering, Zhejiang University, Hangzhou 310027, People's Republic of China

[2]Key Laboratory for Biomedical Engineering of Ministry of Education, College of Biomedical Engineering and Instrument Science, Zhejiang University, Hangzhou 310027, People's Republic of China

[3]Shenzhen Key Laboratory of Laser Engineering, College of Optoelectronic Engineering, Shenzhen University, Shenzhen, 518060, People's Republic of China

[4]College of Chemical and Biological Engineering, Zhejiang University, Hangzhou 310027, People's Republic of China

[5]Ningbo Research Institute, Zhejiang University, Ningbo 315100, People's Republic of China

[6]Ocean College, Zhejiang University, Zhoushan 316021, People's Republic of China

*Corresponding author. yhou@zju.edu.cn (Yang Hou), lujianguo@zju.edu.cn (Jianguo Lu)

Note S1 Measurements and Evaluations

S1.1 Characterizations of 3D hierarchical $NiCo_2O_4$ arrays

Morphologies and structures of the products were characterized by field emission scanning electron microscope (FESEM, Hitachi S-4800) and transmission electron microscope (TEM, FEI F20). Crystal structures were characterized by X-ray diffraction (PANalytical B.V. Empyrean 200895) with a Cu *K*α radiation. The chemical valence states of the products were analyzed by X-ray photoelectron spectroscopy (XPS, Thermo ESCALAB 250) with a monochromatic Al *K*α (1486.6 eV) radiation source.

S1.2 Electrochemical measurements of hierarchical $NiCo_2O_4$ electrodes

Electrochemical measurements of 3D hierarchical $NiCo_2O_4$ arrays were carried out in three-electrode configuration with platinum (Pt) foil (1×2 cm^2) as counter electrode and saturated calomel electrode (SCE) as reference electrode in 3 M KOH aqueous electrolyte. The nickel foam with $NiCo_2O_4$ active materials was used as working electrode directly. The cyclic voltammetry (CV) and galvanostatic charge discharge

(GCD) measurements were conducted as both qualitative and quantitative analysis of electrochemical properties. Electrochemical impedance spectroscopy (EIS) measurements were carried out in a frequency range from 1 Hz to 100 KHz under a sinusoidal disturbance voltage of 5 mV at open circuit potential. The specific capacity (C_1) of working electrode was calculated based on galvanostatic discharge measurements:

$$C_1 = \frac{I_{dis}\Delta t}{m} \tag{S1}$$

where I_{dis} is the discharge current, Δt is the discharging time corresponding to the specified potential change ΔV, and m is the mass of active materials.

S1.3 Evaluations of $NiCo_2O_4$//AC battery-supercapacitor hybrid devices

Electrochemical measurements of 3D hierarchical $NiCo_2O_4$ arrays BSH were carried out in two-electrode system, where cathode ($NiCo_2O_4$), membrane, and anode (AC) are packed. The CV and GCD were conducted in a potential range from 0–1.6 V. The specific capacity (C_2) of BSH was calculated from GCD measurements:

$$C_2 = \frac{I_{dis}\Delta t}{m} \tag{S2}$$

Energy density and power density of BSH were obtained as:

$$E = \frac{i\int vdt}{m} \tag{S3}$$

$$P = \frac{E}{\Delta t} \tag{S4}$$

where v represents the potential.

The constant voltage hold test was carried out as followings. The BSH was charged to 1.6 V at current density of 20 mA cm^{-2} and constantly hold for 5 hours. Then, the device was discharged to 0 V at current density of 20 mA cm^{-2}. The above cycles were repeated up to 100 hours.

S1.4 Evaluations of a-Si:H solar cells

The current density-voltage (J–V) characteristics of *a*-Si:H solar cells were analyzed using a solar simulator (Wacom WXS-140S) at standard test conditions (AM 1.5, 100 mW cm^{-2}, 25°C) with an area of 1.00 cm^2. The external quantum efficiency (EQE) of *a*-Si:H solar cells was calculated from the spectral response measured at zero bias.

S1.5 Evaluations of integrated system

In the integrated system, the *a*-Si:H solar cells were the energy conversion devices, the hierarchical $NiCo_2O_4$ hierarchical arrays BSHs were the energy storage devices, and LEDs were the energy utilization devices. The $NiCo_2O_4$ hierarchical arrays BSHs were charged by the *a*-Si:H solar cells. The LEDs were driven by the $NiCo_2O_4$ hierarchical arrays BSHs. The integrated system was evaluated carefully by the above-mentioned methods.

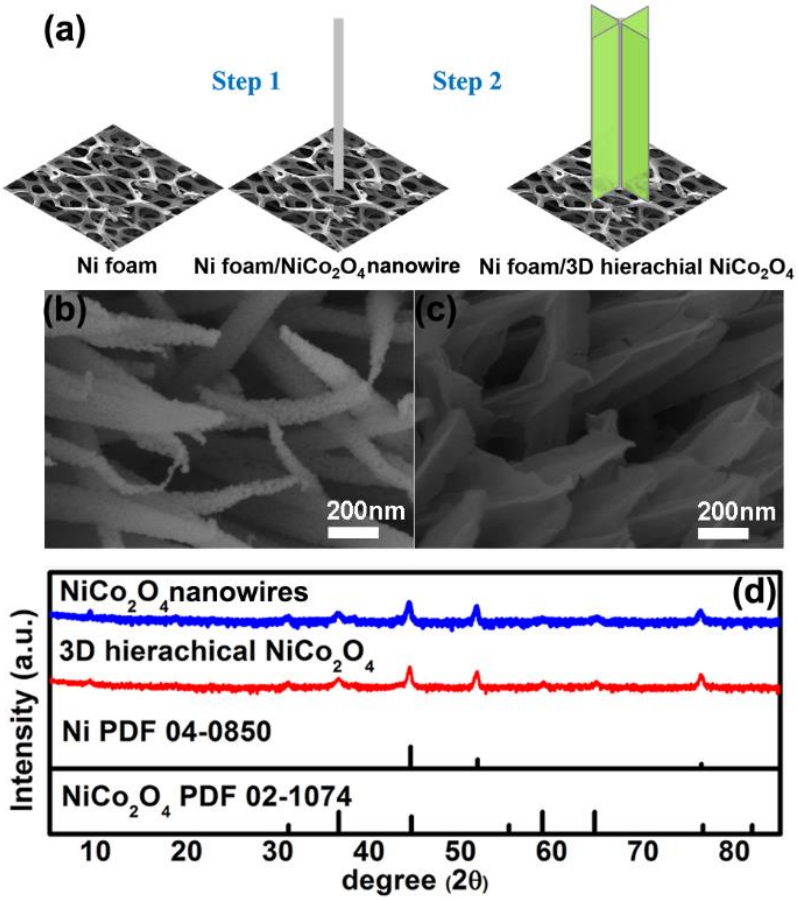

Fig. S1 a Schematic illustration of the formation process of 3D hierarchical $NiCo_2O_4$ arrays. SEM images of **b** $NiCo_2O_4$ nanowire arrays and **c** $NiCo_2O_4$ hierarchical arrays. **d** XRD patterns of $NiCo_2O_4$ nanowire arrays (blue line), $NiCo_2O_4$ hierarchical arrays (red line) and standard diffraction pattern of metallic Ni (PDF 04-0850) and face cubic $NiCo_2O_4$ (PDF 02-1074).

Figure S1a illustrates the experimental scheme of the two-step method for synthesis of 3D hierarchical $NiCo_2O_4$ arrays. SEM was conducted to obtain the morphology of $NiCo_2O_4$ nanowire arrays and finally obtained 3D $NiCo_2O_4$ hierarchical arrays, as shown in Figures S1b and S1c, respectively. The intermediate product of $NiCo_2O_4$ nanowire arrays was successfully grown on the nickel foam, with a shrunk diameter from the bottom to top, and a porous structure with small particles assembled, forming an ideal backbone for the further growth of $NiCo_2O_4$ nanoflakes. After the chemical bath deposition, the final product of 3D hierarchical $NiCo_2O_4$ arrays appears clearly. The 3D hierarchical structure makes all nanowires and nanoflakes highly accessible to electrolyte for energy storage.

To obtain detailed information on the crystal structure, XRD patterns of 3D $NiCo_2O_4$ hierarchical arrays was first conducted as shown in Figure S1d. The main conspicuous peaks located at 44.5°, 51.8°, and 76.4° can be ascribed to metallic nickel (Ni foam). The other five peaks centered at 31.1°, 36.6°, 59.0°, 64.7°, and 76.6° are attributed to the (220), (311), (511), (440), and (553) planes of $NiCo_2O_4$ with face centered cubic crystal. Although the peak at 76.6° of $NiCo_2O_4$ overlaps with the peak at 76.4° of metallic nickel, the other four peaks are consistent well with the standard XRD

patterns of $NiCo_2O_4$, which firmly point out the exact phase structure of our 3D $NiCo_2O_4$ hierarchical arrays.

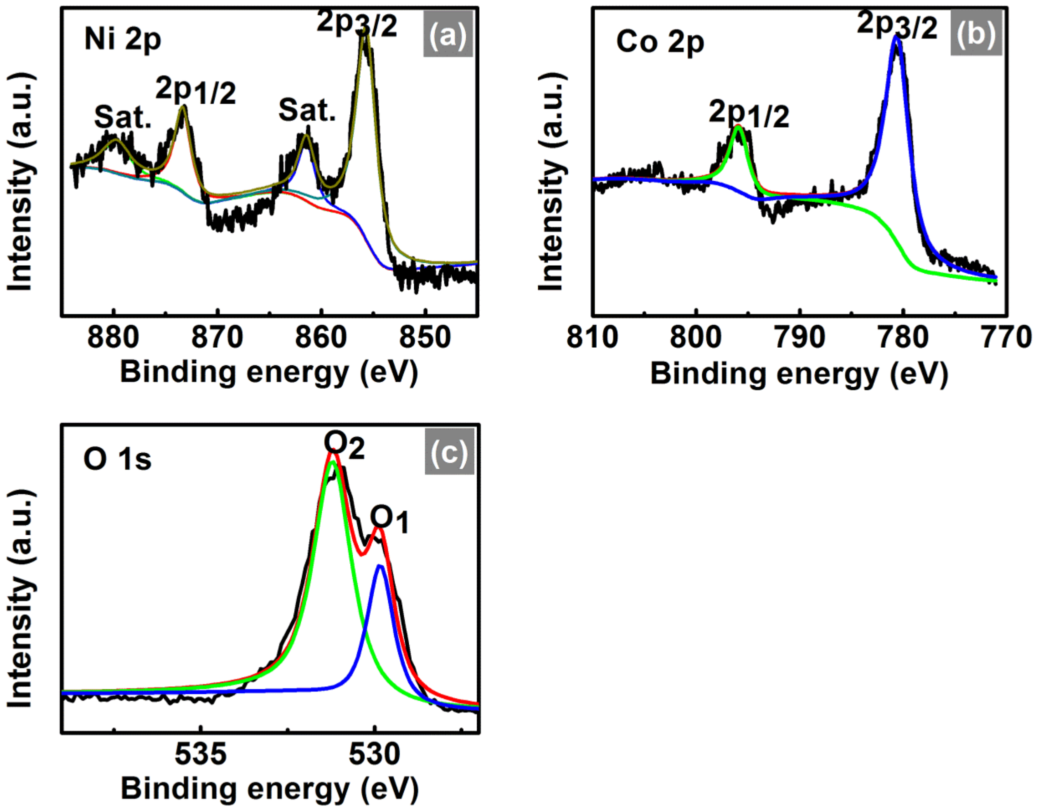

Fig. S2. XPS spectra of **a** Ni 2p, **b** Co 2p, and **c** O 1s of $NiCo_2O_4$ hierarchical arrays

Figures S2a–S2c show the XPS spectra of Ni, Co and O to explore the valence states of elements in $NiCo_2O_4$ arrays. The binding energies of Ni 2p are deconvoluted into four peaks, in which two peaks (873.3 eV of Ni $2p_{1/2}$ and 855.8 eV of Ni $2p_{3/2}$) are assigned to the two spin-orbit doublets of Ni corresponds to characteristic of Ni^{2+} and Ni^{3+}, and the other two peaks (positioned at 879.8 and 861.5 eV) are assigned to two satellites (identified as Sat.) [S1,S2]. Similarly, the Co spectrum is best fitted with two spin-orbit doublets of Co $2p_{3/2}$ (780.7 eV) and Co $2p_{1/2}$ (795.9 eV), corresponding to characteristic of Co^{2+} and Co^{3+}. The O 1s spectrum show two oxygen contributions: the peak positioned at 529.5 eV denoted as O_1 is associated with metal-oxygen bonds in metal oxide, whereas the peak positioned at 531.0 eV (O_2) is corresponding to defect sites with low oxygen coordination in the material with small particle size [S3]. These results show that the as-sythesized sample has a composition of Ni^{2+}, Ni^{3+}, Co^{2+}, Co^{3+}, and metal-bonded O, which is in good agreement with the results in the literature for $NiCo_2O_4$ [S1–S3].

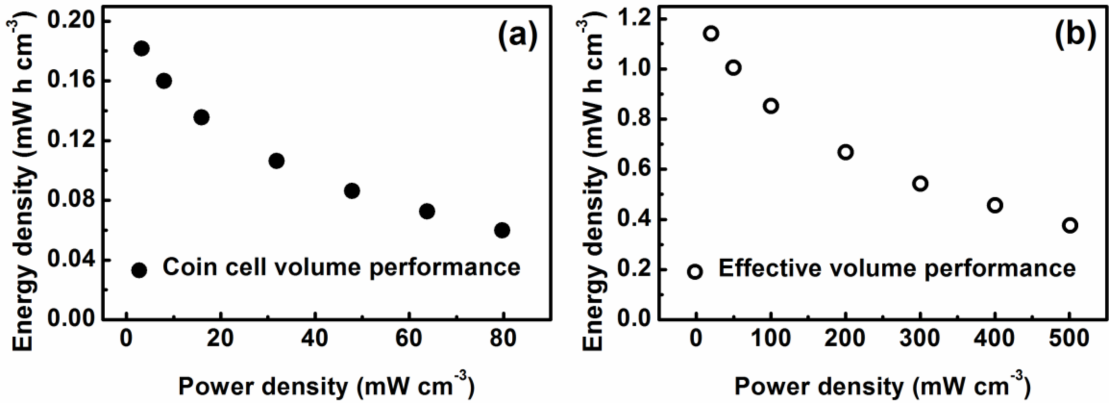

Fig. S3 a Volumetric energy density and power density derived from the volume of a coin cell (CR2016). **b** Volumetric energy density and power density derived from the effective volume

Figure S3a denotes the volumetric performances normalized by coin cell (CR2016) volume, which has a volume of 0.5 cm^3 (with diameter of 2 cm and thickness of 0.16 cm). Figure S3b denotes the volumetric performances normalized by effective volume of electrode and membrane, which has a volume of 0.08 cm^3 (with area of 0.5 cm^2 and thickness of 0.16 cm).
The calculations of coin cell volume and effective volume are provided as:

$$V_{coin} = \pi r^2 h = \pi \times 1 \times 0.16 = 0.5\text{cm}^{-3} \quad (S5)$$

$$V_{effective} = Ah = 0.5 \times 0.16 = 0.08\text{cm}^{-3} \quad (S6)$$

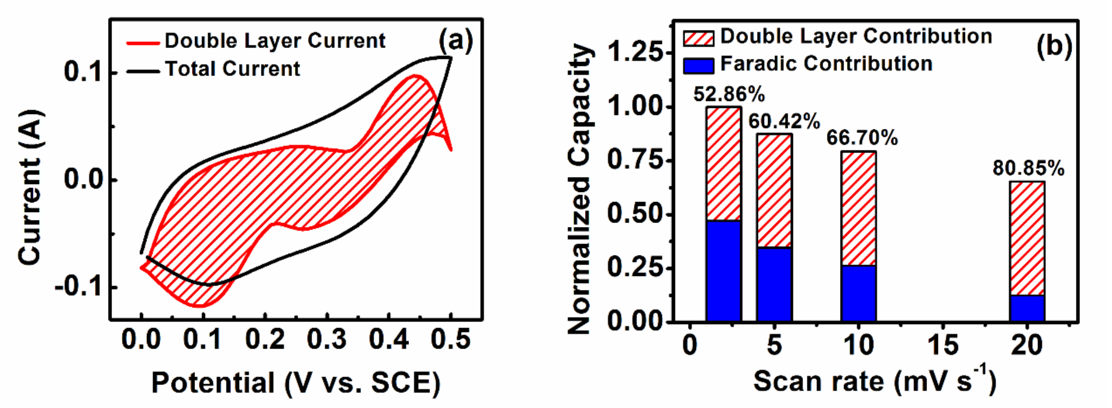

Fig. S4 a CV curve of $NiCo_2O_4$ hierarchical arrays at 20 mV s^{-1} (black line) and calculated double layer current (red line). **b** Charge storage of $NiCo_2O_4$ hierarchical arrays at different scan rate, where the total charge is separated into double layer contribution (red region) and faradic capacity (blue region) and the data indicated in this figure is the percentage of double layer capacity in the whole capacity.

As mentioned in the main text, the $NiCo_2O_4$ hierarchical arrays//AC BSHs exhibit a long cycle lifetime, with 100% retention of capacity after 15000 cycles. No loss is observed in our experiment, which is the evident merit for our BSHs. To the best of our knowledge, this is a pretty long lifetime for supercapacitors with metal oxides and hydroxides as electrodes. To clarify the origin of this stability and the charge storage

mechanism as well, an electrochemical kinetic analysis of $NiCo_2O_4$ hierarchical arrays will be investigated in what follows. In a CV measurement in three electrode configuration, the total charge can be separated into two components: the contributions from surface charge accumulation and the contributions from diffusion controlled intercalation. Therefore, the current response of CV test can be described as the sum of two contributions originated from surface double layer effects and the diffusion controlled intercalation process:

$$i(V) = k_1 v + k_2 v^{0.5} \tag{S7}$$

For analytical purposes, the formula can be rearranged to:

$$i(V) / v^{0.5} = k_1 v^{0.5} + k_2 \tag{S8}$$

In the formula above, $k_1 v$ and $k_2 v$ corresponds to the current contributions from surface double layer effect and the diffusion controlled intercalation, respectively. By determining k_1 and k_2, we are able to quantify the fraction of current contributions from each mechanism.

Figure S4a enables us to determine the fraction of double layer current from the total current response. By comparing the double layer current with the whole current response, we find that the double layer effect contributes only one part to the whole charge storage. Figure S4b shows the total capacity, as well as double layer contribution and intercalation contribution as a function of scan rate. As the scan rate increases, the total capacity of $NiCo_2O_4$ hierarchical arrays decreases. This phenomenon is attributed to the fact that intercalation process can't keep up with the high scan rate due to the slow diffusion of ions. Therefore, only the intercalation part decreases with the scan rate, whereas the double layer contribution keeps almost constant. The data shows that the double layer contribution does not change with the increase of scan rate. The double layer contribution proportions of $NiCo_2O_4$ hierarchical arrays are 52.9%, 60.4%, 66.7%, and 80.9% at the scan rate of 2, 5, 10, and 20 mV s^{-1}, respectively. Clearly, the double layer contribution plays a more and more important role in the whole capacity with the increasing scan rate. Thanks to the high double layer contribution of $NiCo_2O_4$ hierarchical arrays, the whole capacity can keep at a high level under fast charge-discharge conditions. It is believed that the dominant double layer contribution in the whole capacity may be also the reason for the long cycling lifetime.

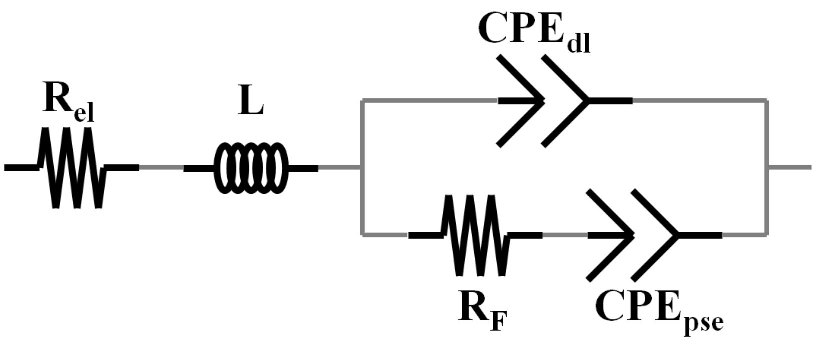

Fig. S5 Equivalent circuit model of EIS fitting

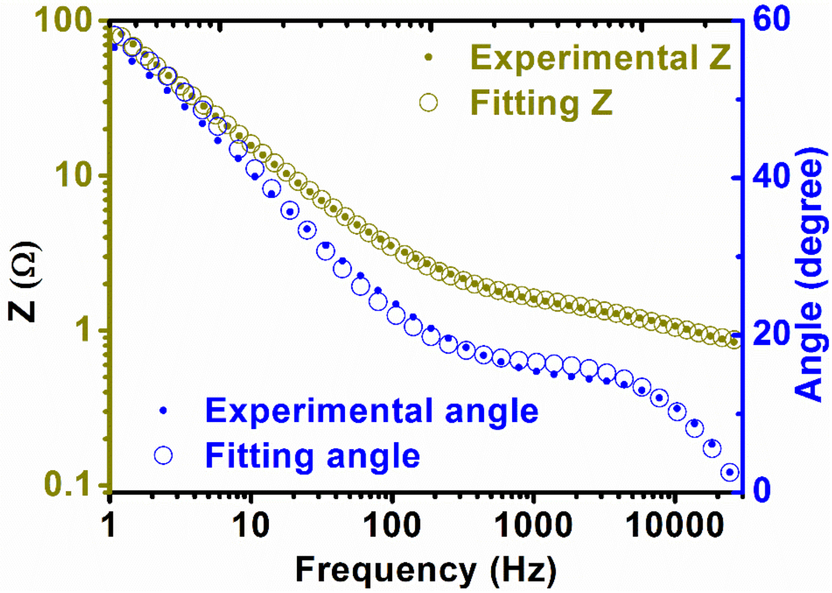

Fig. S6 Bode plot of EIS spectrum of BSH (the filled dots represent experimental data and hollow dots represent fitted data)

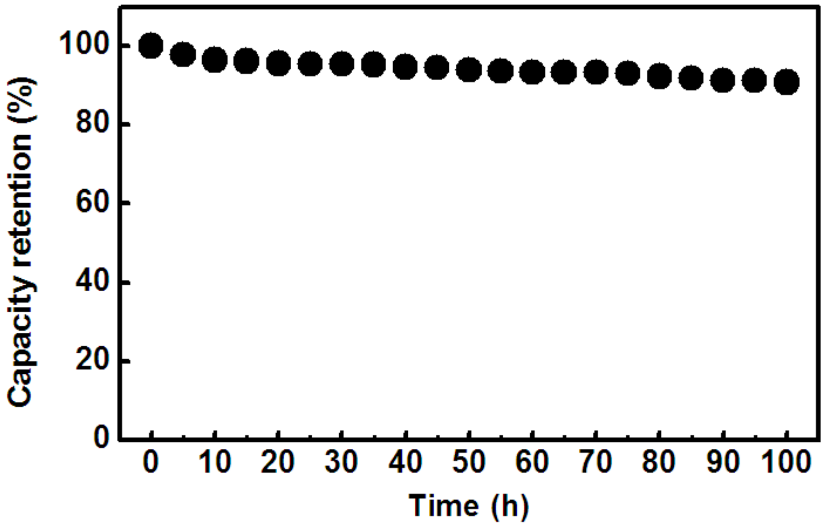

Fig. S7 Constant voltage hold test of $NiCo_2O_4$//AC BSH

The stability of $NiCo_2O_4$//AC BSHs was evaluated by voltage holding test. Specifically, a voltage of 1.6 V was applied to the BSH and charge-discharge was applied every 5 hours between 0 V and 1.6 V with a constant current density of 20 mA cm^{-2}. During a test procedure for 100 hours, the capacity retention of $NiCo_2O_4$//AC was 90.7%. The results further confirm the high stability of the $NiCo_2O_4$//AC BSHs.

Table S1 Mass loading, electrode area, effective volume and volume of coin cell of the BSH devices

Cathode ($NiCo_2O_4$)	Mass loading (mg cm^{-2})	3.3
	Area (cm^2)	0.333
Anode (active carbon)	Mass loading (mg cm^{-2})	8.8
	Area (cm^2)	0.5
**Effective Volume**	Effective area (cm^2)	**0.5**
	Overall thickness (cm)	**0.16**
	Effective Volume (cm^3)	**0.08**
**Coin cell (CR2016)**	Thickness (cm)	**0.16**
	Diameter (cm)	**2.0**
	Volume (cm^3)	**0.50**

We propose two "volume" here. The "effective volume" denotes the real volume of active materials, including cathode, anode and membrane. The coin cell volume is the volume of cell we used to package our active materials. It should be noted that the volume of coin cell is much larger than the effective volume in this study; that's why we propose two "volume" here with two different definitions. Coin cell volume is the actual volume in our study, but effective volume reflects the real volume of active material with reference significance and represents real volumetric performance if the package process is optimized.

Table S2 Overall efficiency and storage efficiency of photo voltage-energy storage systems in almost all the available literatures reported in recent years.

year	Energy storage module (Supercapacitors (SCs))	η (%)	Storage efficiency (%)	literature
2007	Li-polymer-battery	1.85	80	[S4]
2012	Ti//CNT fiber	1.5	68.4	[S5]
2013	PANI//PANI	2.1	46	[S6]
2013	RuO_2//RuO_2 SCs	0.8	26.67	[S7]
2014	Ni(Co)O//AC SCs	0.6	12.24	[S8]
2014	Ti@TiO_2//CNT SCs	1.2	75.7	[S9]
2014	Ti@TiO_2//MWCNT SCs	0.82	65.6	[S10]
2015	Bacterial cellulose membrane/polypyrrole nanofibers/MWCNTs// Bacterial cellulose membrane/polypyrrole nanofibers/MWCNTs SCs	10	73.53	[S11]
2016	Carbon//carbon SCs	2.9	46.77	[S12]
2016	PEDOT//carbon SCs	4.7	73.78	[S13]
2017	Polypyrrole//polypyrrole SCs	10.5	78.42	[S14]
2017	MnO_2//carbon SCs	5.26	67.52	[S15]
2018	Carbon//carbon SCs	7.1	79.78	[S16]
	$NiCo_2O_4$//active carbon SCs	8.1	74.24	This work

References

[S1] G. Li, W. Y. Li, K. B. Xu, R. J. Zou, Z. G. Chen, J. Q. Hu, Sponge-like $NiCo_2O_4/MnO_2$ ultrathin nanoflakes for supercapacitor with high-rate performance and ultra-long cycle life. J. Mater. Chem. A **2**(21), 7738-7741 (2014). https://doi.org/10.1039/c4ta01337a

[S2] B. Cui, H. Lin, Y.-z. Liu, J.-b. Li, P. Sun, X.-c. Zhao, C.-j. Liu, Photophysical and photocatalytic properties of core-ring structured $NiCo_2O_4$ nanoplatelets. J. Phys. Chem. C **113**(32), 14083-14087 (2009). https://doi.org/10.1021/jp900028t

[S3] J. F. Marco, J. R. Gancedo, M. Gracia, J. L. Gautier, E. Ríos, F. J. Berry, Characterization of the nickel cobaltite, $NiCo_2O_4$, prepared by several methods: An xrd, xanes, exafs, and xps study. J. Solid State Electrochem. **153**(1), 74-81 (2000). https://doi.org/10.1006/jssc.2000.8749

[S4] G. Dennler, S. Bereznev, D. Fichou, K. Holl, D. Ilic, R. Koeppe, M. Krebs, A. Labouret, C. Lungenschmied, A. Marchenko, D. Meissner, E. Mellikov, J. Méot, A. Meyer, T. Meyer, H. Neugebauer, A. Öpik, N. S. Sariciftci, S. Taillemite, T. Wöhrle, A self-rechargeable and flexible polymer solar battery. Solar Energy **81**(8), 947-957 (2007). https://doi.org/10.1016/j.solener.2007.02.008

[S5] T. Chen, L. Qiu, Z. Yang, Z. Cai, J. Ren, H. Li, H. Lin, X. Sun, H. Peng. An integrated "energy wire" for both photoelectric conversion and energy storage. Angew. Chem. Int. Ed. **51**(48), 11977-11980 (2012). https://doi.org/10.1002/anie.201207023

[S6] Y. Fu, H. Wu, S. Ye, X. Cai, X. Yu, S. Hou, H. Kafafy, D. Zou, Integrated power fiber for energy conversion and storage. Energy Environ. Sci. **6**(3), 805 (2013). https://doi.org/10.1039/c3ee23970e

[S7] M. Skunik-Nuckowska, K. Grzejszczyk, P. J. Kulesza, L. Yang, N. Vlachopoulos, L. Häggman, E. Johansson, A. Hagfeldt, Integration of solid-state dye-sensitized solar cell with metal oxide charge storage material into photoelectrochemical capacitor. J. Power Sources **234**, 91-99 (2013). https://doi.org/10.1016/j.jpowsour.2013.01.101

[S8] N. Bagheri, A. Aghaei, M. Y. Ghotbi, E. Marzbanrad, N. Vlachopoulos, L. Häggman, M. Wang, G. Boschloo, A. Hagfeldt, M. Skunik-Nuckowska, P. J. Kulesza, Combination of asymmetric supercapacitor utilizing activated carbon and nickel oxide with cobalt polypyridyl-based dye-sensitized solar cell. Electrochim. Acta **143**, 390-397 (2014). https://doi.org/10.1016/j.electacta.2014.07.125

[S9] X. Chen, H. Sun, Z. Yang, G. Guan, Z. Zhang, L. Qiu, H. Peng, A novel “energy fiber” by coaxially integrating dye-sensitized solar cell and electrochemical capacitor. J. Mater. Chem. A **2**(6), 1897-1902 (2014). https://doi.org/10.1039/c3ta13712k

[S10] Z. Zhang, X. Chen, P. Chen, G. Guan, L. Qiu, H. Lin, Z. Yang, W. Bai, Y. Luo, H. Peng, Integrated polymer solar cell and electrochemical supercapacitor in a flexible and stable fiber format. Adv. Mater. **26**(3), 466-470 (2014). https://doi.org/10.1002/adma.201302951

[S11] X. Xu, S. Li, H. Zhang, Y. Shen, S. M. Zakeeruddin, M. Graetzel, Y. B. Cheng, M. Wang, A power pack based on organometallic perovskite solar cell and supercapacitor. ACS Nano **9**(2), 1782-1787 (2015). https://doi.org/10.1021/nn506651m

[S12] B. P. Lechêne, M. Cowell, A. Pierre, J. W. Evans, P. K. Wright, A. C. Arias, Organic solar cells and fully printed super-capacitors optimized for indoor light

energy harvesting. Nano Energy **26**, 631-640 (2016). https://doi.org/10.1016/j.nanoen.2016.06.017

[S13] J. Xu, Z. Ku, Y. Zhang, D. Chao, H. J. Fan, Integrated photo-supercapacitor based on PEDOT modified printable perovskite solar cell. Adv. Mater. Technol. **1**(5), 1600074 (2016). https://doi.org/10.1002/admt.201600074

[S14] R. Liu, J. Wang, T. Sun, M. Wang, C. Wu, H. Zou, T. Song, X. Zhang, S. T. Lee, Z. L. Wang, B. Sun, Silicon nanowire/polymer hybrid solar cell-supercapacitor: A self-charging power unit with a total efficiency of 10.5. Nano Lett. **17**(7), 4240-4247 (2017). https://doi.org/10.1021/acs.nanolett.7b01154

[S15] Z. Liu, Y. Zhong, B. Sun, X. Liu, J. Han, T. Shi, Z. Tang, G. Liao, Novel integration of perovskite solar cell and supercapacitor based on carbon electrode for hybridizing energy conversion and storage. ACS Appl. Mater. Interfaces **9**(27), 22361-22368 (2017). https://doi.org/10.1021/acsami.7b01471

[S16] J. Liang, G. Zhu, Z. Lu, P. Zhao, C. Wang, Y. Ma, Z. Xu, Y. Wang, Y. Hu, L. Ma, T. Chen, Z. Tie, J. Liu, Z. Jin, Integrated perovskite solar capacitors with high energy conversion efficiency and fast photo-charging rate. J. Mater. Chem. A **6**(5), 2047-2052 (2018). https://doi.org/10.1039/c7ta09099d
